# The issue beyond resistance: Methicillin-resistant *Staphylococcus epidermidis* biofilm formation is induced by subinhibitory concentrations of cloxacillin, cefazolin, and clindamycin

**DOI:** 10.1371/journal.pone.0277287

**Published:** 2022-11-09

**Authors:** Rasoul Mirzaei, Rasoul Yousefimashouf, Mohammad Reza Arabestani, Iraj Sedighi, Mohammad Yousef Alikhani

**Affiliations:** 1 Department of Microbiology, School of Medicine, Hamadan University of Medical Sciences, Hamadan, Iran; 2 Department of Pediatrics, School of Medicine, Hamadan University of Medical Sciences, Hamadan, Iran; 3 Brucellosis Research Center, Hamadan University of Medical Sciences, Hamadan, Iran; Thomas Jefferson University, UNITED STATES

## Abstract

*Staphylococcus epidermis* is one of the most frequent causes of device-associated infections due to biofilm formation. Current reports noted that subinhibitory concentrations of antibiotics induce biofilm production in some bacteria. Accordingly, we evaluated the effect of exposure of different subinhibitory concentrations of cloxacillin, cefazolin, clindamycin, and vancomycin on the biofilm formation of methicillin-resistant *S*. *epidermidis* (MRSE). Antimicrobial susceptibility testing and minimum inhibitory/bactericidal concentration of antimicrobial agents were determined. MRSE isolates were selected, and their biofilm formation ability was evaluated. The effect of subinhibitory concentrations of cloxacillin, cefazolin, clindamycin, and vancomycin, antibiotics selected among common choices in the clinic, on MRSE biofilm formation was determined by the microtitre method. Besides, the effect of subinhibitory concentrations of cloxacillin, cefazolin, clindamycin, and vancomycin on the expression of the biofilm-associated genes *icaA* and *atlE* was evaluated by Reverse-transcription quantitative real-time polymerase chain reaction (RT-qPCR). Antimicrobial susceptibility patterns of MRSE strains showed a high level of resistance as follows: 80%, 53.3%, 33.3%, 33.3%, and 26.6%, for erythromycin, trimethoprim-sulfamethoxazole, tetracycline, clindamycin, and gentamicin, respectively. Besides, 73.3% of *S*. *epidermidis* strains were Multidrug-resistant (MDR). Minimum inhibitory concentration (MIC) and minimum bactericidal concentration (MBC) values were in the range of 0.5 to512 μg/mL and 1 to1024 μg/mL for cloxacillin, 0.125 to256 μg/mL and 1 to512 μg/mL for cefazolin, 0.125 to64 μg/mL and 4 to>1024 μg/mL for clindamycin, and 2 to32 μg/mL and 4 to32 μg/mL for vancomycin, respectively. The findings showed that subinhibitory concentrations of cloxacillin, cefazolin, and clindamycin induce biofilm production in MRSE strains. In particular, the OD values of strains were in the range of 0.09–0.95, 0.05–0.86, and 0.06–1 toward cloxacillin, cefazolin, and clindamycin, respectively. On the other hand, exposure to subinhibitory vancomycin concentrations did not increase the biofilm formation in MRSE strains. The findings also demonstrated that sub-MIC of antibiotics up-regulated biofilm-associated genes. In particular, *atlE* and *icaA* were up-regulated 0.062 to 1.16 and 0.078 to 1.48 folds, respectively, for cloxacillin, 0.11 to 0.8, and 0.1 to 1.3 folds for cefazolin, 0.18 to 0.98, and 0.19 to 1.4 folds, respectively, for clindamycin. In contrast, the results showed that sub-MIC of vancomycin did not increase the biofilm-associated genes. These findings overall show that exposure to sub-MIC of traditional antibiotics can cause biofilm induction in MRSE, thereby increasing the survival and persistence on various surfaces that worsen the condition of comorbid infections.

## 1. Introduction

Antibiotics since their discovery have been applied with excellent success in the treatment and prevention of infections and have saved thousands of millions of lives [1]. Nevertheless, the overuse of these antimicrobial agents has led to the emergence of pan-drug-resistant (PDR) bacteria due to improper and inappropriate use, which indicates the post-antibiotic era [2]. In this regard, it was estimated that 10 million people will die each year from drug-resistant bacterial infections by 2050, but this prediction has changed due to the devastating effect of the Coronavirus disease 2019 (COVID-19) pandemic on the over usage of antibiotics in healthcare systems [3, 4]. Along with the direct threat of COVID-19 infection to patients, increased use of disinfectants, including hand sanitizers and surface cleaners, is anticipated to cause increased rates of antimicrobial resistance in pathogenic bacteria in the coming years [5]. Researchers in recent years studied some of these issues, calling for a ban on over-the-counter antibiotic sales and strict protocols for the prescription of antibiotics by physicians [6]. On top of this, antibiotic overuse can be associated with less well-known phenomenon’s changes in the bacteria, and a growing body of reports suggests that bacteria exposed to inappropriate antibiotics could stimulate the formation of some factors such as the toxin, adhesin, as well as biofilm [7–9]. In this scenario, biofilms are a complex structure of bacteria embedded in extracellular polymeric substances (EPS) composed of proteins, extracellular DNA (eDNA), and polysaccharides [10–12]. This mode of bacterial life protects embedded bacterial biofilm from dehydration, antimicrobial molecules, and immune cells [13, 14]. According to reports, approximately 80% of bacterial infections are related to biofilm, which is extremely hard to cure [13, 14]. Importantly, in most cases, bacteria are present as the biofilm to further colonization of the skin, oropharyngeal, nose, and intestine, as well as indwelling devices like implants and central venous catheters (CVCs) [15]. In these environments, the bacteria are guided to produce biofilm by environmental signals, many of which have been recognized and some of which are unknown [15].

Bacterial biofilm increases antibiotic tolerance and resistance by 100–1000 folds than planktonic cells [16, 17]. Besides, in some cases, the concentration of the antibiotic given to the biofilm infection site cannot achieve its optimal inhibitory or bactericidal levels, and the bacterial cells are exposed to sub-inhibitory concentrations [18, 19]. Several reports show that sub-inhibitory concentrations of antimicrobial agents can change bacteria’s physiological and biochemical functions [18, 20]. In this regard, current records noted how the sub-MIC concentrations of antibiotics could induce stress in bacteria, causing a selection of the more resistant cells and, in some bacterial species inducing biofilm production [21, 22]. In this regard, Jin et al. [21] found that sub-MIC values of mupirocin cause biofilm production in *Staphylococcus aureus*. In pioneer works, induction of the biofilm-related genes was found in *Staphylococcus epidermidis* to subinhibitory concentrations of erythromycin [23, 24]. Methicillin-resistant *S*. *epidermidis* (MRSE) causes device-associated infections due to the potential for biofilm production [17, 25–27].

Beta-lactams are among the most commonly used antibiotics to treat staphylococcal infections [28]. Although a high prevalence of MRSE and beta-lactam resistant populations have been found, the biofilm-inducing effect of methicillins/beta-lactams on MRSE is unknown while this biofilm-inducing effect was demonstrated on MRSA [28]. Besides, it has been found that clindamycin, which is used in the treatment of Gram-positive infections, triggers the *S*. *aureus* biofilm formation at subinhibitory concentrations [29]. Most patients who get empiric antibiotics for sepsis before the offending organism is identified receive beta-lactam antibiotics plus vancomycin, and it is probable that this stimulates MRSE biofilm development and limits vancomycin efficacy in some individuals [28]. More importantly, it is clear that host tissues, wounds, and biofilm-infected medical equipment are exposed to a variety of antibiotic concentrations throughout antibiotic therapy [30]. Accordingly, in the current study, we evaluated the effects of different subinhibitory concentrations of cloxacillin, cefazolin, clindamycin, and vancomycin, antibiotics selected among common choices in the clinic, on inducing biofilm formation of MRSE.

## 2. Methods

### 2.1. Ethical statement

This investigation was approved by Hamadan University of Medical Sciences, Hamadan, Iran (Ethics No. IR.UMSHA.REC.1398.571).

### 2.2. Media, chemical reagents, and antibiotics

The antibiotics disks were obtained from MAST (Mast Co., UK). Cloxacillin, cefazolin, clindamycin, and vancomycin powders were obtained from Sigma-Aldrich Company (USA). Mannitol salt agar, Blood agar, Mueller-Hinton agar, DNA agar, Mueller-Hinton broth (MHB), and NaCl were obtained from Merck (Merck Company, USA). Crystal violet, glucose, and agarose were obtained from Sigma-Aldrich Company (USA). U-Bottom 96 well sterile polystyrene microplates were obtained from NEST Co., Ltd, China.

### 2.3. Collection, phenotypic and genotypic tests for detection *Staphylococcus epidermidis* strains

From April to August 2019, 159 *Staphylococcus* isolates were taken from hospitalized patients at wards of hospitals in Hamedan, Iran. All *Staphylococcus* isolates were directly cultured on blood agar overnight at 37 °C [31]. *S*. *epidermidis* was recognized by biochemical tests such as the gram-staining, catalase reaction, resistance to bacitracin and polymyxin B, coagulase reaction, sensitivity to novobiocin, and mannitol fermentation [32]. In addition, molecular confirmation of *S*. *epidermidis* was performed via species-specific primers by polymerase chain reaction (PCR) [33]. *S*. *epidermidis* ATCC 35984 was kindly provided by Dr. Eyup Dogan (Ankara, Turkey). *S*. *epidermidis* DSMZ 3270 was kindly provided by Prof. Bibi Sedigheh Fazly Bazzaz (Mashhad, Iran). Additionally, *S*. *aureus* ATCC 25923 and *S*. *aureus* ATCC 29213 were purchased from the Pasteur Culture Collection of Iran, Tehran, Iran.

### 2.4. Detection of MRSE, antibacterial susceptibility testing, and MDR

In the present study, *S*. *epidermidis* isolates were studied for their resistance to methicillin by phenotypic method, i.e., cefoxitin (30 μg) and oxacillin (1 μg) via the Kirby-Bauer method based on the guideline of the Clinical and Laboratory Standards Institute (CLSI) [34]. Then, in phenotypically detected MRSE isolates, the existence of the *mecA* gene was studied by PCR via previously designed primers [35]. In brief, the DNA of *S*. *epidermidis* was extracted by using the DNA Purification kit (Roche Co., Berlin, Germany) based on the manufacturer’s instructions. Then, the PCR mixture was prepared in a 25 μL volume containing 1.5 μL MgCl_2_, 2.5 μL PCR buffer (10X), 1 μL of Taq polymerase (5 U; Ampliqon Co., Herlev, Denmark), 0.6 μl dNTP (10 mmol/l), 2 μl of extracted DNA, 1.4 μL forward and reverse primers, as well as 16 μL distilled water (DW). The DNA amplification was conducted in a thermal cycler (Eppendorf Co., Hamburg, Germany) via the following conditions: denaturation at 95°C for 5 min, 30 cycles with denaturation at 95 °C for 2 min, annealing at 60°C for 1 min, extension at 70°C for 1 min and followed by a final extension at 72°C for 10 min [36]. Finally, PCR products were visualized by 1% agarose gel in Tris/Borate/EDTA buffer for 40 min via the gel documentation system.

In the following, 15 *S*. *epidermidis* strains, including clinical MRSE, and ATCC strains, were used for further evaluation. In this regard, to determine of susceptibility pattern of these selected *S*. *epidermidis* strains, the Kirby-Bauer method was done based on CLSI recommendations for the following disks: gentamicin (10 μg), erythromycin (15 μg), clindamycin (2 μg), tetracycline (30 μg), linezolid (30 μg), and trimethoprim-sulfamethoxazole (1.25 μg). *S*. *aureus* ATCC 25923 was applied as quality control [34]. The *S*. *epidermidis* strains that were resistant to at least one agent in ≥ three classes of antimicrobial agents were considered multidrug-resistant (MDR) [37].

### 2.5. Minimum inhibitory concentration

It should be noted that in the current study cloxacillin, cefazolin, clindamycin, and vancomycin antibiotics representative of various common antibiotics in the clinic for the treatment of *S*. *epidermidis* infections were selected. Micro broth dilution assay was applied to determine the minimum inhibitory concentration (MIC) to cloxacillin, cefazolin, clindamycin, and vancomycin against *S*. *epidermidis* strains based on the CLSI recommendations [34]. Briefly, fresh *S*. *epidermidis* colonies were cultured in MHB, shaking at 180 rpm overnight at 37 °C. The next day, the number of bacterial cells was set to 0.5 McFarland turbidity using a spectrophotometer at 625 nm. It should be noted that, in the current study, to enhance the accuracy of the bacterial count, the optical density (OD) of the bacterial suspension was set to 0.09. Then, the number of provided bacterial suspensions was set to 10^6^ colony-forming units (CFUs)/mL by dilution at the same medium. At the same time, serial dilutions of cloxacillin, cefazolin, clindamycin, and vancomycin were prepared in the wells by MHB at a volume of 100 μL in a 96-well microplate. The amount of cloxacillin, cefazolin, clindamycin, and vancomycin was ranged from 0.25 to 1024 μg/mL, 0.125 to 1024 μg/mL, 0.125 to 1024 μg/mL, and 0.5 to 64 μg/mL, respectively. Finally, 100 μL of prepared bacterial suspension equal to 10^5^ CFUs were inoculated with each of the diluted antibiotics, and the microplate was incubated for 18–24 h at 37 °C based on the tested antibiotic. The lowest antibiotic concentration causes complete inhibition of bacterial growth visibly was chosen as MIC based on the CLSI definition [34]. MIC assays were repeated at least 3 times for all strains.

### 2.6. Minimum bactericidal concentration and MBC/MIC value

MBC for cloxacillin, cefazolin, clindamycin, and vancomycin were measured according to the CLSI recommendations [34]. In brief, fresh *S*. *epidermidis* colonies were cultured in MHB with shaking at 180 rpm overnight at 37 °C, and the next day the number of bacteria was set to 0.5 McFarland turbidity, and then, 10^6^ CFUs/mL as described above. At the same time, serial dilution of the tested antibiotic was prepared in the wells at a volume of 100 μL in a 96-well microplate as described above. Finally, 100 μL from the provided bacterial suspension equal to 10^5^ CFUs were inoculated into wells of the diluted antibiotic as described above, and the microplate was incubated overnight at 37 °C. The next day, 10 μL from each well was cultured on MHA, and the grown colonies were counted. Based on the definition, the MBC for cloxacillin, cefazolin, clindamycin, and vancomycin were considered the lowest value of tested antibiotics that killed 100% of the cultured bacteria [38].

Finally, the MBC/MIC value was calculated to characterize the tolerance in selected *S*. *epidermidis* strains. In this regard, *S*. *epidermidis* tolerance against antibiotics was considered if MBC/MIC value ≥32 or MBC/MIC value ≥16 when the MBC was ≥ the breakpoint for bacterial resistance [39].

### 2.7. Screening of biofilm-forming in *Staphylococcus epidermidis* isolates

Among selected *S*. *epidermidis* strains, the ability of biofilm production was evaluated by microtiter plate assay in a Treated, U-Bottom, 96 well microplates as previously described with some changes [40]. Briefly, *S*. *epidermidis* fresh colonies were cultured in 5 mL TSB supplemented with 1% glucose (1% Glu TSB), shaking at 180 rpm overnight at 37 °C. On the next day, 0.5 McFarland turbidity was prepared by measuring the absorbance of the bacterial suspension at a wavelength of 625 nm. Then, 100 μL from the prepared bacterial suspension equal to 10^7^ CFUs was added to 900 μL 1% Glu TSB in a sterile tube, and finally, 200 μL from this prepared suspension equal to 2×10^6^ CFUs was added to each well of 96 well microplates and incubated at 37 °C for 24 h at 75 rpm. Afterward, the contents of the wells were gently drained, and wells were gently washed three times with normal saline and then air-dried. In the next step, 200 μL absolute methanol was entered into each well to fixation of produced biofilm, and after 15 min, the contents of the wells were drained, and the wells were air-dried again. Then, the wells were stained with 200 μL of 0.05% crystal violet for 5 min, the solution was drained, and the wells were washed three times with normal saline and air-dried again. Finally, 200 μL of absolute ethanol was entreated to each well with shaking for 30 min at 37 °C, and finally, the content of each well was moved in to new well in a new microplate and, ultimately, the absorbance was determined at 595 nm using a Synergy^™^ HTX Multi-Mode Microplate Reader (BioTek Co., Winooski, USA). 1% Glu TSB without bacterial cells was applied as the negative control, and *S*. *epidermidis* ATCC 35984 was used as the positive control for biofilm production. The capability of the tested *S*. *epidermidis* strain to biofilm formation was categorized as follows: OD ≤ OD cut-off (ODc), non-biofilm forming; ODc < OD ≤ 2 × ODc, weak biofilm-forming; 2 × ODc < OD ≤ 4 × ODc, moderate biofilm-forming; and 4 × ODc < OD, strong biofilm-forming [17, 37]. ODc was considered as three standard deviations (SDs) above the mean OD of the negative control [37]. The biofilm formation assay was repeated three times.

### 2.8. Effect of sub-MIC values of cloxacillin, cefazolin, clindamycin, and vancomycin on inducing biofilm formation

A micro broth dilution assay was applied to determine the sub-MIC concentrations as mentioned above. In brief, fresh *S*. *epidermidis* colonies were cultured in 5 mL of MHB at 37 °C with shaking at 180 rpm for 24 h. Then, the number of bacterial cells was set to 0.5 McFarland turbidity, as described above. In the next step, sub-MIC concentrations acquired from MIC determination of antibiotics ranged from 0.125 to 128 μg/mL for cloxacillin, 0.0312 to 16 μg/mL for clindamycin, 0.0312 to 128 μg/mL for cefazolin, and 0.25 to 16 μg/mL for vancomycin were prepared on 96 well microplates. It should be noted that the volume of antibiotics on the wells was 100 μL per well. Then, 100 μL suspension of tested bacterium equal to 2×10^6^ CFUs was added to each well, and the microplate was incubated at 37 °C for 24 h with shaking at 75 rpm. After overnight incubation, the content of the wells was gently aspirated and washed 3 times with normal saline solution. Finally, the quantity of formed biofilm was measured by microtiter plate assay, as explained above. Each experiment was performed in triplicate for all *S*. *epidermidis* strains. TSB+1% Glu and MHB without antibiotics were applied as positive and negative controls, respectively.

### 2.9. Effect of sub-inhibitory values of cloxacillin, cefazolin, clindamycin, and vancomycin on the expression of the biofilm-associated genes

#### 2.9.1. RNA extraction and cDNA synthesis

*S*. *epidermidis* strains include four methicillin-resistant, and ATCC 35984 was selected to evaluate biofilm-associated expression genes, i.e., *atlE* and *icaA*, by real-time PCR. The selected strains were exposed with sub-MIC concentrations of antibiotics ranging from 0.125 to 128 μg/mL for cloxacillin, 0.0312 to 16 μg/mL for clindamycin, 0.0312 to 128 μg/mL for cefazolin, and 0.25 to 16 μg/mL for vancomycin for 24 h. Then, the mRNA was extracted by RNA extraction Kit (Gene All Co., South Korea), based on the manufacturer’s instructions. It should be noted that the RNA concentration, purity, and integrity were checked by the NanoDrop spectrophotometer (Thermo Scientific Co., USA) and the agarose gel, respectively. A total of 1 μg RNA was applied for cDNA synthesis by a Two-step RT-PCR Kit based on the manufacturer’s instruction (Gene All Co., South Korea).

#### 2.9.2. Quantitative real-time PCR

The expression of biofilm-associated genes was quantified by the 2X Q-PCR Master Mix (SYBR, no ROX) (SMOBIO, Taiwan) containing 2μL cDNA and 1μL from each primer of *atlE*, *icaA*, and *16S rRNA* genes in a final volume of 20 μL on the RT-PCR system (LightCycler^®^ 96 Instrument, Roche Co., USA). The *atlE*, *icaA*, and *16S rRNA* primers were used in previous studies [41]. The *16S rRNA* gene was amplified as the internal control. In this test, the reaction condition was as follows: initial denaturation at 95 °C for 10 min, followed by 40 cycles of 95 °C for 15 sec, annealing at 60 °C for 45 sec, and extension at 72 °C for 30 sec. After PCR cycling, melting point findings were collected, and a dissociation curve was evaluated for each well. To control amplification efficiency, the standard curve was designed for the *atlE* and *icaA* by serial dilutions of isolated RNA from untreated *S*. *epidermidis* ATCC 35984. Besides, the gene expression was calculated by the ΔΔCt method [42]. To find up-regulated and down-regulated genes, log Fold-changes were calculated in the log2 scale (equal to ΔΔCt) that log2 > 0 indicates up-regulation and log2 < 0 indicates down-regulation of genes [43, 44].

### 2.10. Statistical analysis

The GraphPad Prism (version 9) was applied to all statistical analyses. MIC and MBC values for each antibiotic were done at least three times. Most of the findings are generally expressed as the mean and standard deviation (SD) unless otherwise indicated. Besides, a paired-sample t-test was applied to determine the significance of the findings from each concentration of tested antibiotic in terms of the amount of biofilm formation and gene expression in treated and non-treated groups. Additionally, a one-way analysis of variance (ANOVA) was used to compare the differences in expression of the biofilm-associated genes between the various treated concentrations and the control. The findings were expressed as the mean ± SD. The normality testing (Gaussian distribution) for data was checked and passed by Shapiro-Wilk and Kolmogorov-Smirnov tests. Additionally, appropriate test (t-test) was used for two groups and results reported. Besides, for doing a t-test such as the scale of measurement, random sampling, normality of data distribution, adequacy of sample size, and equality of variance in standard deviation were confirmed. To use the ANOVA test we confirmed: each group sample was drawn from a normally distributed population, all populations had a common variance, and all samples were drawn independently of each other. Additionally, Tukey’s post-hoc test for compare the mean of each group with the mean of each other group was done. All of the analyses were performed with a confidence level of 95%. Besides, P-values < 0.05 were considered statistically significant.

## 3. Results

### 3.1. Phenotypic and genotypic tests for detection of *S*. *epidermidis* and MRSE

In the present study, 97 isolates were characterized as *S*. *epidermidis* by biochemical tests, and all of these isolates were confirmed as *S*. *epidermidis* by PCR. These *S*. *epidermidis* isolates were collected from blood (n = 46), catheter (n = 17), wound (n = 15), urine (n = 14), and sputum (n = 5). The result of antibacterial susceptibility testing and molecular test of *S*. *epidermidis* isolates toward cefoxitin, oxacillin, and *mecA* gene showed that 72.1% (n = 70) of *S*. *epidermidis* isolates were detected as MRSE. In particular, 78.2% (n = 36), 73.3% (n = 11), 64.2% (n = 9), 64.7% (n = 11), 60% (n = 3) of blood, wound, urine, catheter, and sputum, respectively were methicillin resistant. Finally, 13 MRSE clinical isolates were selected based on the survey of resistance to methicillin, that is, isolates with higher resistance (i.e. a smaller inhibition zone for cefoxitin and oxacillin) were selected and with *S*. *epidermidis* ATCC 35984 and *S*. *epidermidis* DSMZ 3270 were used for further study.

### 3.2. Antibacterial susceptibility testing and determination of MDR *S*. *epidermidis* strains

The disk diffusion data showed the antibiotic resistance rate of *S*. *epidermidis* strains toward erythromycin, trimethoprim-sulfamethoxazole, tetracycline, clindamycin, and gentamicin 80%, 53.3%, 33.3%, 33.3%, and 26.6%, respectively. By and large, 73.3% (n = 11) strains of *S*. *epidermidis* were MDR. Further details of antibacterial susceptibility testing among strains are shown in Table 1.

**Table 1:** The results of antimicrobial susceptibility testing in *Staphylococcus epidermidis* strains.

Strains (n = 15)	FOX	OX	E	TS	TE	CD	GM	MDR/NonMDR
**ATCC 35984**	R	R	R	R	R	R	I	MDR
**DSMZ 3270**	S	S	S	S	S	S	S	NonMDR
**MRSE 1**	R	R	R	R	S	R	R	MDR
**MRSE 2**	R	R	R	R	S	R	R	MDR
**MRSE 3**	R	R	S	S	S	S	S	NonMDR
**MRSE 4**	R	R	I	S	S	S	R	NonMDR
**MRSE 5**	R	R	R	R	S	R	R	MDR
**MRSE 6**	R	R	R	R	R	R	S	MDR
**MRSE 7**	R	R	R	R	S	S	S	MDR
**MRSE 8**	R	R	R	R	R	S	S	MDR
**MRSE 9**	R	R	R	S	S	S	S	MDR
**MRSE 10**	R	R	R	R	R	S	S	MDR
**MRSE 11**	R	R	R	S	S	S	S	MDR
**MRSE 12**	R	R	R	S	R	S	S	MDR
**MRSE 13**	R	R	R	S	S	I	S	NonMDR

Abbreviations: ATCC, American Type Culture Collection; DSMZ, Deutsche Sammlung von Mikroorganismen und Zellkulturen; MRSE, methicillin-resistant *Staphylococcus epidermidis*; R, resistant; I, intermediate; S, sensitive; FOX, cefoxitin; OX, Oxacillin; E, Erythromycin; TS, Trimethoprim-Sulfamethoxazole; TE, Tetracycline; CD, Clindamycin; GM, Gentamicin; MDR, multidrug-resistant.

### 3.3. MIC, MBC, and MBC/MIC value

**Fig 1:**
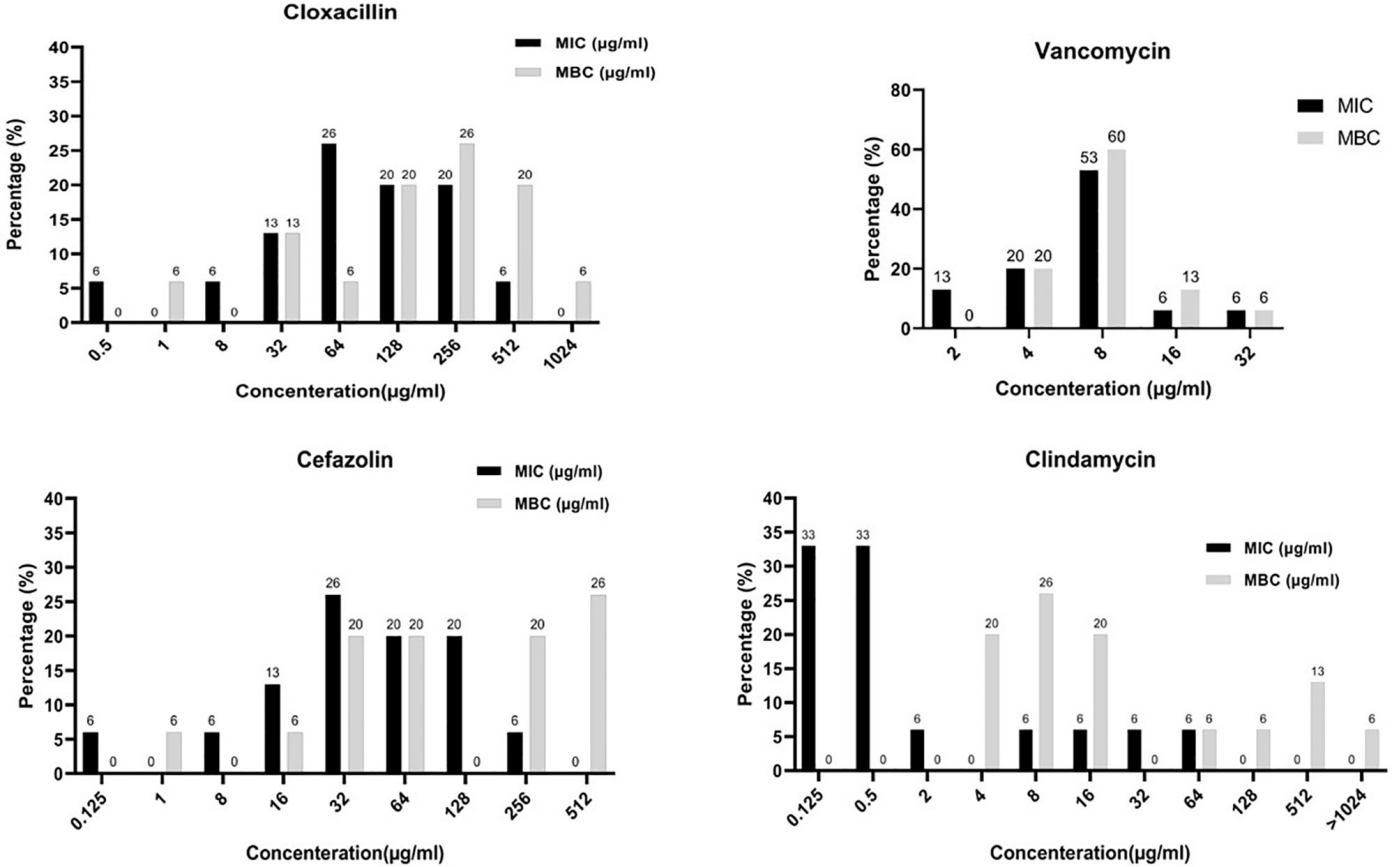
Frequency distribution of MIC and MBC of cloxacillin, cefazolin, clindamycin, and vancomycin toward *Staphylococcus epidermidis* strains. Reference to the results, the growth of the majority of strains was prevented at lower amounts of vancomycin in comparison to cefazolin, clindamycin and cloxacillin. Vancomycin at the amount of 32 μg/mL prevented the growth of all the examined strains. In contrast, cefazolin, clindamycin and cloxacillin at the concentration of 256, 64 and 512 μg/mL, respectively, were able to prevent the growth of the strains. MIC, minimum inhibitory concentration; MBC, minimum bactericidal concentration. Y-axis is the percentage of frequency distribution of MIC and MBC.

The geometric means of the MIC of cloxacillin, cefazolin, clindamycin, and vancomycin against *S*. *epidermidis* strains were 64, 32, 0.95, and 6.64 μg/mL, respectively. Besides, the geometric means of the MBC of cloxacillin, cefazolin, clindamycin, and vancomycin against *S*. *epidermidis* strains were 134.05, 88.44, 26.59, and 8.37 μg/mL, respectively. The MIC_50_ for cloxacillin, cefazolin, clindamycin, and vancomycin were 64, 32, 0.5, and 8 μg/mL, and MBC_50_ cloxacillin, cefazolin, clindamycin, and vancomycin were 256, 64, 16, and 8 μg/mL, respectively. Besides, the MIC_90_ for cloxacillin, cefazolin, clindamycin, and vancomycin were 256, 128, 32, and 16 μg/mL, and also, MBC_90_ for cloxacillin, cefazolin, clindamycin, and vancomycin were 512, 512, 512, and 16 μg/mL, respectively. Additionally, one vancomycin-resistant MDR-MRSE with MIC equal to 32 μg/mL was found. The geometric mean of MBC/MIC for cloxacillin, cefazolin, clindamycin, and vancomycin against stains was 2.09, 2.76, 28.98, and 1.25 μg/mL. Further details are shown in Table 2 and Fig 1.

**Table 2:** Minimum inhibitory concentration and minimum bactericidal concentration of cloxacillin, cefazolin, clindamycin, and vancomycin against *Staphylococcus epidermidis*.

Strains	CLO-MIC (μg/mL)	CLOMBC (μg/mL)	CLO-MBC/MIC ratio	CD-MIC(μg/mL)	CD-MBC (μg/mL)	CD-MBC/MIC ratio	CEF-MIC (μg/mL)	CEF-MBC(μg/mL)	CEF-MBC/MIC ratio	Van-MIC (μg/mL)	Van-MBC (μg/mL)	Van-MBC/MIC ratio
**ATCC 35984**	64	256	4	8	128	16	32	32	1	2	4	2
**DSMZ 3270**	0.5	1	2	0.5	16	32	0.125	1	8	2	4	2
**MRSE 1**	64	128	2	16	512	32	32	64	2	16	16	1
**MRSE 2**	32	32	1	32	512	16	32	32	1	8	8	1
**MRSE 3**	128	256	2	0.125	8	64	64	256	4	4	8	2
**MRSE 4**	128	256	2	0.5	4	8	32	64	2	8	8	1
**MRSE 5**	256	512	2	0.125	8	64	128	512	4	32	32	1
**MRSE 6**	512	1024	2	64	>1024	>16	256	512	2	8	8	1
**MRSE 7**	64	128	2	0.5	16	32	64	256	4	8	8	1
**MRSE 8**	256	512	2	0.125	4	32	128	512	4	8	8	1
**MRSE 9**	32	128	4	0.125	8	64	16	32	2	8	16	2
**MRSE 10**	128	256	2	0.5	16	32	64	256	4	8	8	1
**MRSE 11**	64	64	1	0.5	8	16	16	64	4	4	8	2
**MRSE 12**	8	32	4	0.125	4	32	8	16	2	8	8	1
**MRSE 13**	256	512	2	2	64	32	128	512	4	4	4	1
**GM-MIC**	64	-	-	0.95	-	-	32	-	-	6.64	-	-
**GM-MBC**	-	134.05	-	-	26.59	-	-	88.44	-	-	8.37	-
**MIC 50**	64	-	-	0.5	-	-	32	-	-	8	-	-
**MBC 50**	-	256	-	-	16	-	-	64	-	-	8	-
**MIC 90**	256	-	-	32	-	-	128	-	-	16	-	-
**MBC 90**	-	512	-	-	512	-	-	512	-	-	16	-

Abbreviations: ATCC, American Type Culture Collection; DSMZ, Deutsche Sammlung von Mikroorganismen und Zellkulturen; MRSE, methicillin-resistant *Staphylococcus epidermidis*; CEF, cefazolin; CLO, cloxacillin; Van, vancomycin; CD, clindamycin; MIC, minimum inhibitory concentration; MBC, minimum bactericidal concentration; GM-MIC, geometric mean-MIC; GM-MBC, geometric mean-MBC.

### 3.4. Biofilm formation assay

The findings showed that all selected *S*. *epidermidis* strains could form biofilm. The maximum and minimum OD values of biofilm formation for strains induced by 1% Glu TSB were 3.1 and 0.1, respectively. According to the findings, the biofilm formation capability of the *S*. *epidermidis* isolates was classified as strong and moderate producers. Further details are shown in the S1 Table in S1 File.

### 3.5. Effect of sub-MIC values of antibiotics on inducing biofilm production

**Fig 2:**
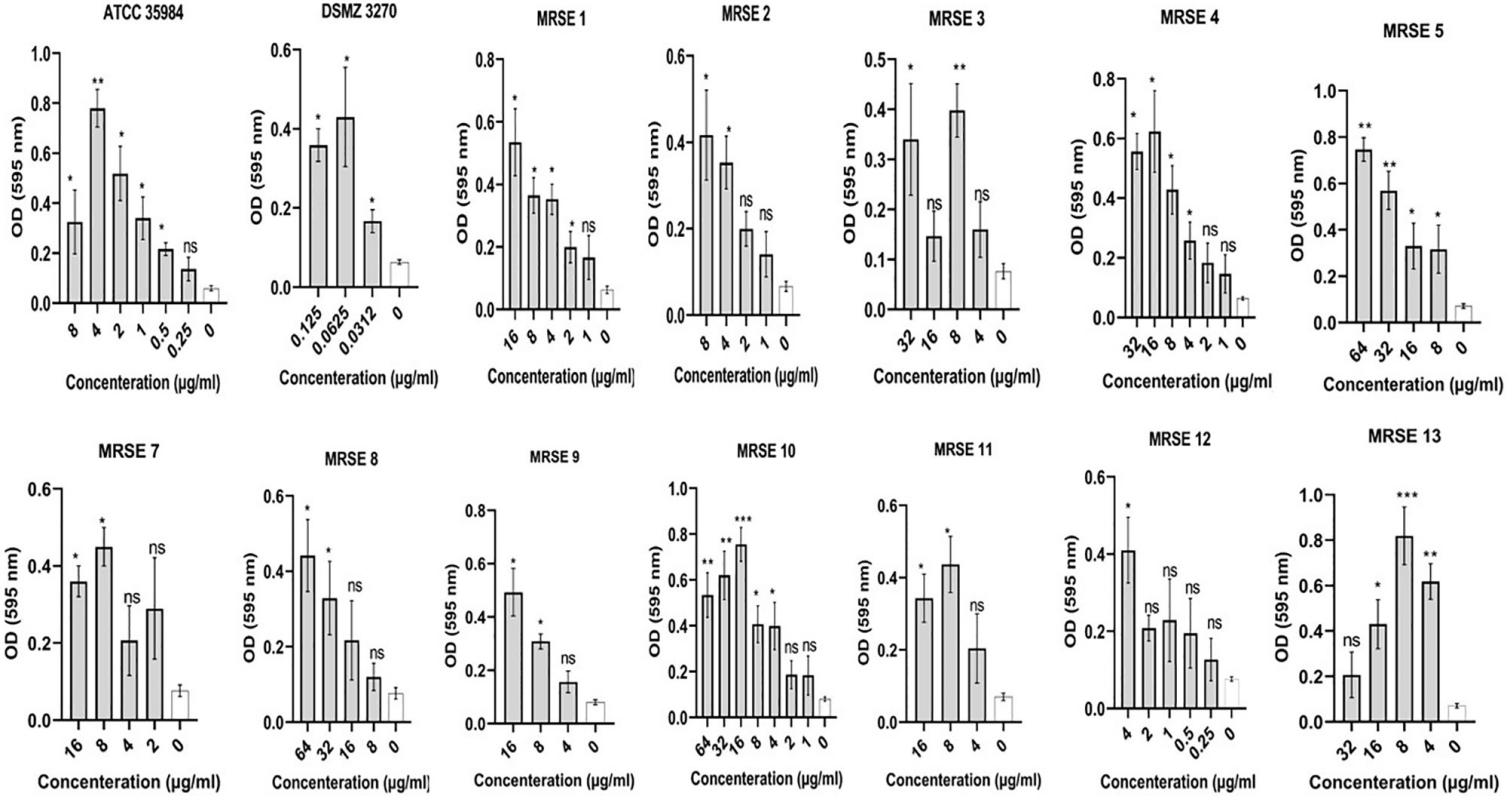
Graphical depiction of the biofilm production (OD values) of each *Staphylococcus epidermidis* strain exposed to sub-MIC concentrations of cloxacillin. In total, the OD values of strains were in the range of 0.09–0.95 and maximum OD increase was found at concentration of 4 μg for ATCC 35984. NS, non-significant; *p < 0.05 and **p ≤ 0.01; OD, Optical density; sub-MIC, sub-minimum inhibitory concentration. Three repeats were performed as depicted on the graphs. Error bars represent standard deviations. T-test was used to compare the significant difference between the untreated (negative) control and exposed state of strain. The asterisks are in relation to bar 0 (untreated state of strain). Data represent the mean ± SD for 3 different experiments for and 14 samples.

**Fig 3:**
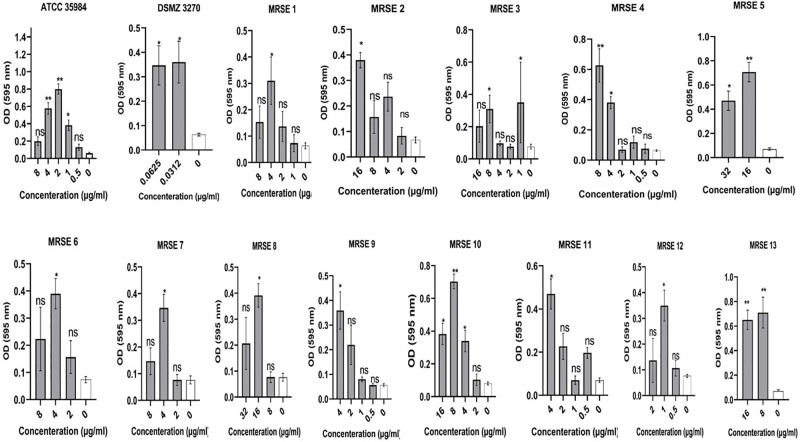
Graphical depiction of the biofilm production (OD values) of *Staphylococcus epidermidis* strain exposed to sub-MIC concentrations of cefazolin. In total, the OD values of strains were in the range of 0.05–0.86 and maximum OD increase was found at concentration of 2 μg for ATCC 35984. NS, non-significant; *p < 0.05 and **p ≤ 0.01; OD, Optical density; sub-MIC, sub-minimum inhibitory concentration. Three repeats were performed as depicted on the graphs. Error bars represent standard deviations. T-test was used to compare the significant difference between the untreated (negative) control and exposed state of strain. The asterisks are in relation to bar 0 (untreated state of strain). Data represent the mean ± SD for 3 different experiments for and 15 samples.

**Fig 4:**
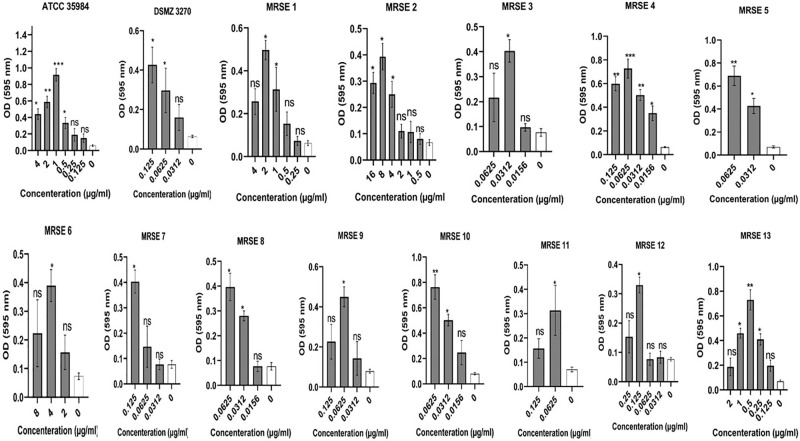
Graphical depiction of the biofilm production (OD values) of *Staphylococcus epidermidis* strain exposed to sub-MIC concentrations of clindamycin. In total, the OD values of strains were in the range of 0.06–1 and maximum OD increase was found at concentration of 1 μg for ATCC 35984. NS, non-significant; *p < 0.05 and **p ≤ 0.01; OD, Optical density; sub-MIC, sub-minimum inhibitory concentration. Three repeats were performed as depicted on the graphs. Error bars represent standard deviations. T-test was used to compare the significant difference between the untreated (negative) control and exposed state of strain. The asterisks are in relation to bar 0 (untreated state of strain). Data represent the mean ± SD for 3 different experiments for and 15 samples.

**Fig 5:**
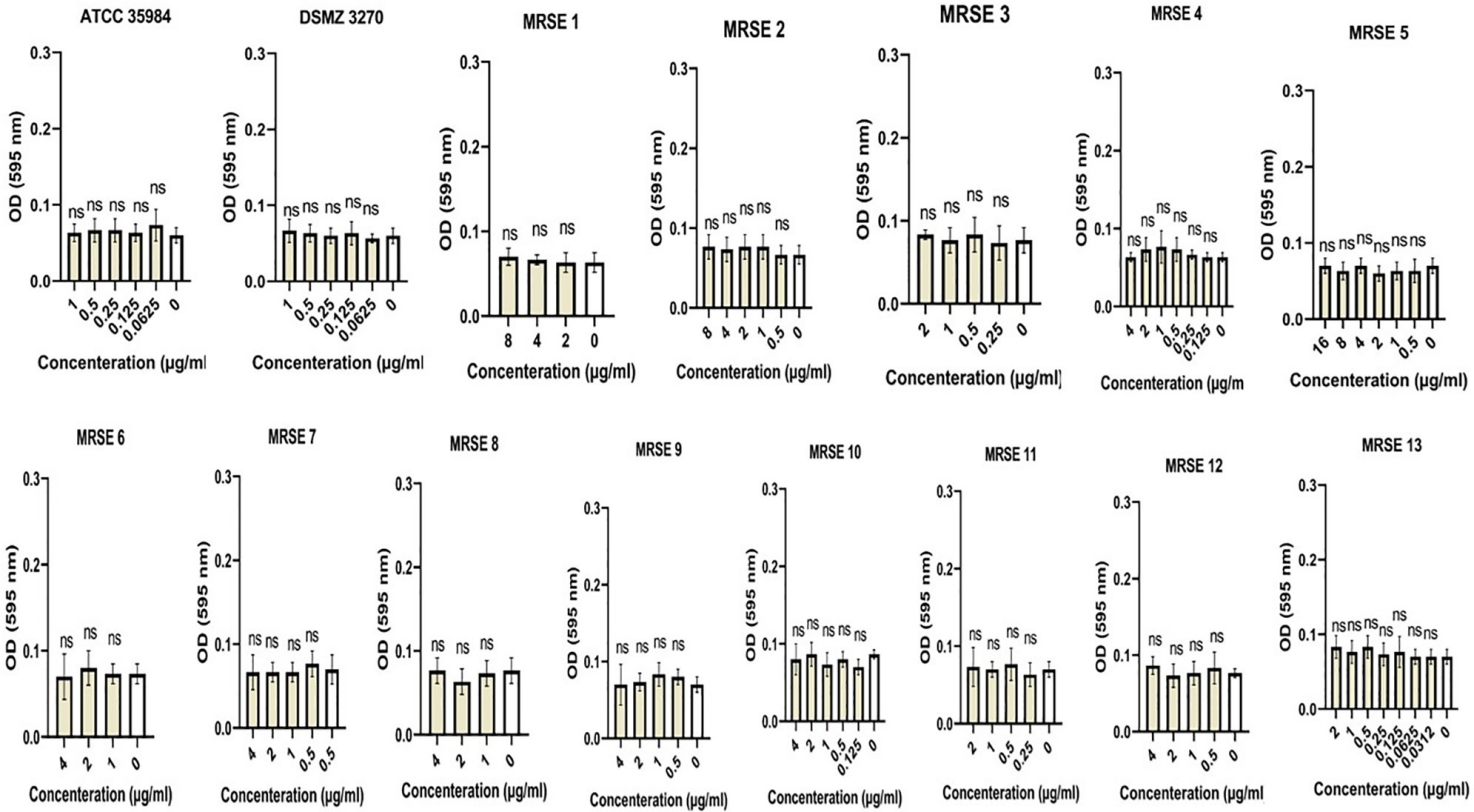
Graphical depiction of the biofilm production (OD values) of *Staphylococcus epidermidis* strain exposed to sub-MIC concentrations of vancomycin. NS, non-significant; OD, Optical density; sub-MIC, sub-minimum inhibitory concentration. Three repeats were performed as depicted on the graphs. Error bars represent standard deviations. T-test was used to compare the significant difference between the untreated (negative) control and exposed state of strain. The asterisks are in relation to bar 0 (untreated state of strain). Data represent the mean ± SD for 3 different experiments for and 15 samples.

Sub-MIC concentrations of cloxacillin, cefazolin, clindamycin, and vancomycin on biofilm production of clinical MRSE and ATCC strains were evaluated. Any increase in OD value, representing a rise in the number of adhered bacterial cells, was considered to enhance biofilm production as described for routine biofilm formation assay. The findings showed that subinhibitory concentrations of cloxacillin, cefazolin, and clindamycin induced biofilm formation in several strains. In particular, the OD values of strains were in the range of 0.09–0.95, 0.05–0.86, and 0.06–1 toward cloxacillin, cefazolin, and clindamycin, respectively. Maximum OD increase for cloxacillin was found at concentration of 4 μg for ATCC 35984. Besides, maximum OD increase for cefazolin was found at concentration of 2 μg for ATCC 35984. Finally, maximum OD increase for clindamycin was found at concentration of 1 μg for ATCC 35984. On the other hand, MRSE and ATCC strains did not increase biofilm formation toward subinhibitory vancomycin concentrations. Further details are shown in Figs 2–5 and the S2 Table in S1 File.

### 3.6. Effect of sub-MIC values of antibiotics on expression of the biofilm-associated genes

After phenotypic analysis of increased biofilm production in the face of sub-MIC concentrations of antibiotics, ATCC 35984, MRSE 4, MRSE5, MRSE 10, and MRSE 13 showing the highest increase in OD were selected for expression of *atlE* and *icaA* genes by RT-PCR. The results showed that expression of *atlE* and *icaA* genes were up-regulated in examined *S*. *epidermidis* strains against cloxacillin, cefazolin, and clindamycin at some concentrations. *AtlE* and *icaA* were up-regulated 0.062±0.04 to 1.16±0.25 and 0.078±0.02 to 1.48±0.12 folds, respectively, at a range of 64 to 0.25 μg of cloxacillin. In this regard, maximum *atlE* and *icaA* upregulation equal to 1.22±0.3 for 64 μg (P value = 0.0193) and 1.49±0.15 for 32 μg (P value = 0.0026) was found in MRSE 5, and MRSE 10. Besides, *atlE* and *icaA* were up-regulated 0.11±0.8 to 0.8±0.24 and 0.1±0.54 to 1.3±0.6 folds, respectively, at the range 32 to 0.5 μg cefazolin. In this regard, maximum *atlE* and *icaA* upregulation equal to 0.82±0.3 for 2 μg (P value = 0.0063) and 1.25±0.25 for 8 μg (P value = 0.0071) was found in ATCC 35984, and MRSE 10.

**Fig 6:**
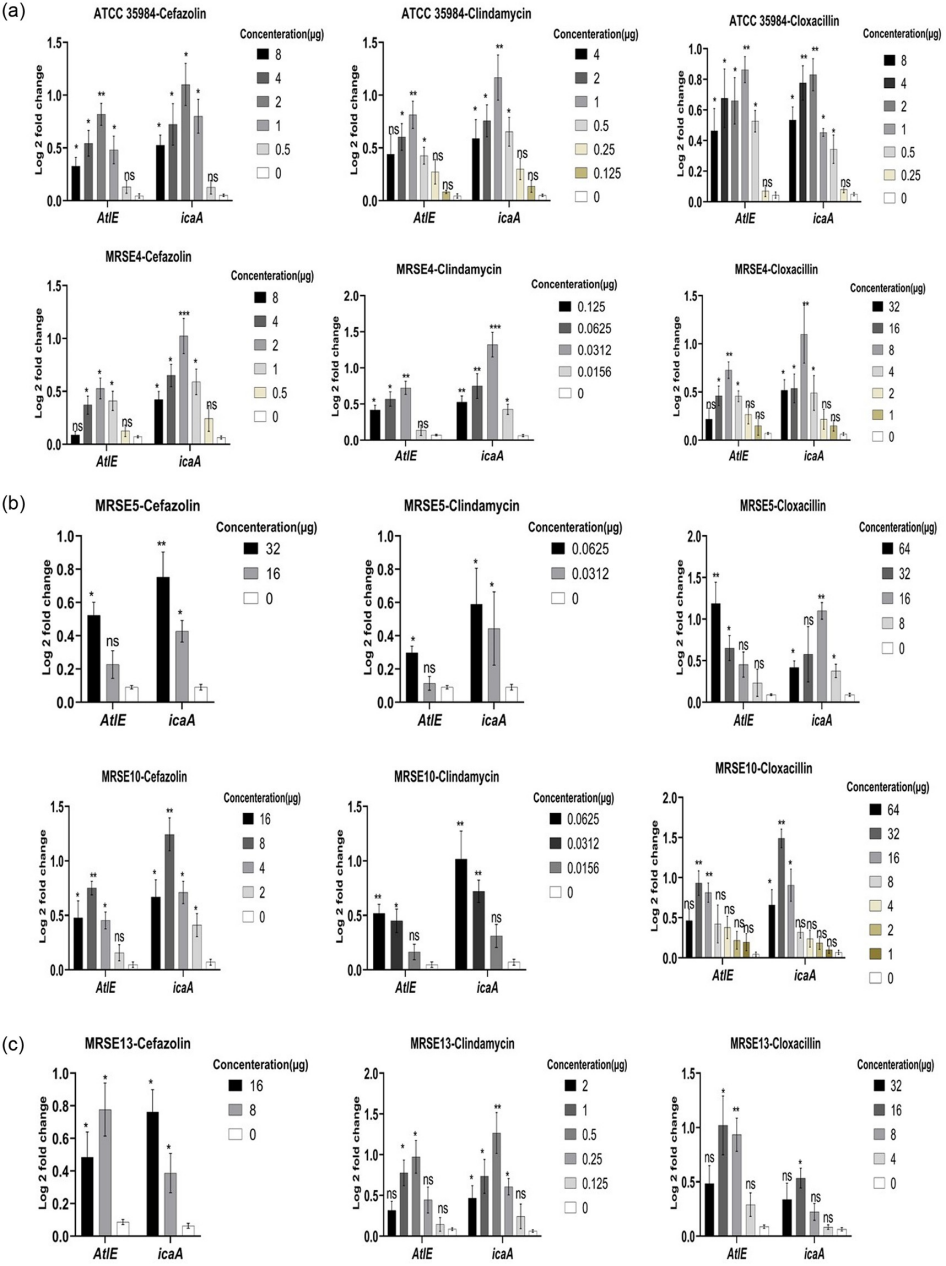
Up-regulation of biofilm-associated genes in *Staphylococcus epidermidis* encounter sub-MIC concentrations of cloxacillin, cefazolin, and clindamycin. *AtlE* and *icaA* were up-regulated 0.062±0.04 to 1.16±0.25 and 0.078±0.02 to 1.48±0.12 folds, respectively, at a range of 64 to 0.25 μg of cloxacillin. Besides, *atlE* and *icaA* were up-regulated 0.11±0.8 to 0.8±0.24 and 0.1±0.54 to 1.3±0.6 folds, respectively, at the range 32 to 0.5 μg cefazolin. Finally, *atlE* and *icaA* were up-regulated 0.18±0.08 to 0.98±0.4 and 0.19±0.24 to 1.4±0.3 folds, respectively, at range 4 to 0.0156 μg clindamycin. The findings are expressed as the mean ± SD. ANOVA indicated a significant difference between various sub-MIC concentrations and control (p-value < 0.05). NS, non-significant; *p < 0.05 and **p ≤ 0.01. Sub-MIC, sub-minimum inhibitory concentration. Three repeats were performed as depicted on the graphs. Error bars represent standard deviations. Data represent the mean ± SD for 3 different experiments for and 5 samples.

**Fig 7:**
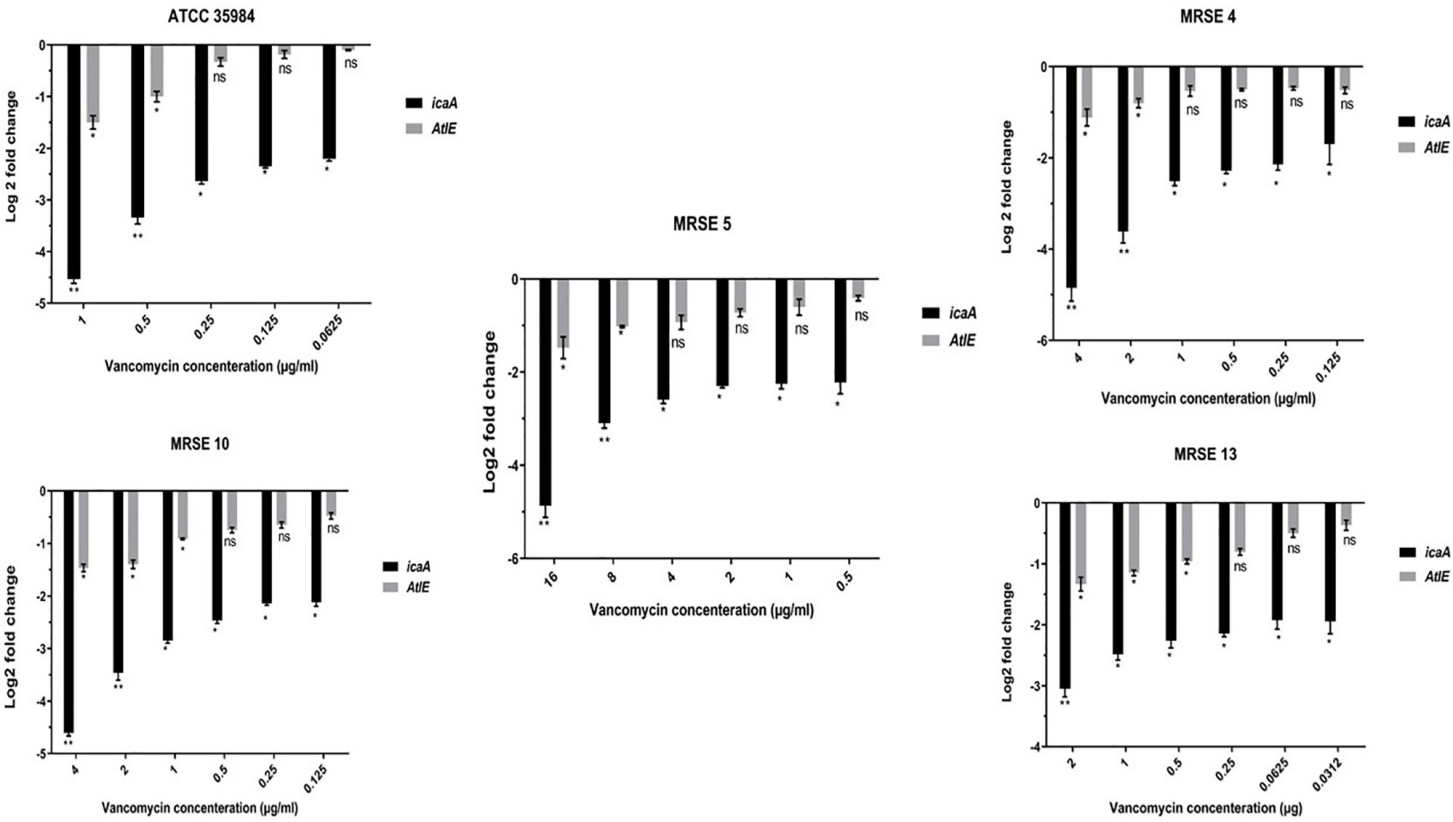
Downregulation of biofilm-associated genes in 5 selected *Staphylococcus epidermis* strains at different sub-MIC concentrations of vancomycin. *AtlE* and *icaA* genes were down-regulated 0.5±0.25 to 1.35±0.34 and 2.11±0.35 to 4.6±0.87 folds, respectively, at a range of 16 to 0.25 μg vancomycin. NS, non-significant; *p < 0.05 and **p ≤ 0.01. The findings are expressed as the mean ± SD. sub-MIC, sub-minimum inhibitory concentration. Three repeats were performed as depicted on the graphs. Error bars represent standard deviations. Data represent the mean ± SD for 3 different experiments for and 5 samples.

Finally, *atlE* and *icaA* were up-regulated 0.18±0.08 to 0.98±0.4 and 0.19±0.24 to 1.4±0.3 folds, respectively, at range 4 to 0.0156 μg clindamycin. In this regard, maximum *atlE* and *icaA* upregulation equal to 1.07±0.18 for 0.5 μg (P value = 0.0145) and 1.35±0.16 for 0.5 μg (P value = 0.0134) was found in MRSE 13. Further details are depicted in Fig 6. ANOVA showed a significant difference between the sub-MIC biofilm-inducing concentrations and negative control for the expression of genes (p< 0.05). On the opposite side, *atlE* and *icaA* genes were down-regulated 0.5±0.25 to 1.35±0.34 and 2.11±0.35 to 4.6±0.87 folds, respectively, at a range of 16 to 0.25 μg vancomycin. Further details are depicted in Fig 7.

## 4. Discussion

*S*. *epidermidis* is one of the most frequent causes of device-associated infections due to the potential of biofilm production on indwelling medical devices [27, 45–47]. Compared to planktonic cells, *S*. *epidermidis* biofilm is known to be more resistant to antibiotics and human immune reactions [48, 49]. In *S*. *epidermidis*, methicillin resistance is an additional vital factor to consider as a nosocomial bacterial pathogen, and on top of this, MDR-MRSE strains drastically limit the therapeutic options available and represent a crucial challenge for human health [50–53]. In the present study, antimicrobial susceptibility patterns of MRSE strains showed a high level of resistance as follows: 80%, 53.3%, 33.3%, 33.3%, and 26.6%, for erythromycin, trimethoprim-sulfamethoxazole, tetracycline, clindamycin, and gentamicin, respectively. Our results were the same as Najar-Peerayeh et al. [54], who reported erythromycin as the most common antimicrobial agent that *S*. *epidermidis* strains were resistant to. Of note, in the present study, 73.3% of strains were MDR which is higher than of others that a possible reason for this discrepancy could be the inclusion criteria for the selection of isolates in our study because we selected methicillin-resistant *S*. *epidermidis* isolates [36, 55]. High levels of methicillin resistance in staphylococci have led to the use of antibiotics such as vancomycin; nevertheless, resistance to this vancomycin has been found in Coagulase-negative staphylococci (CoNS) [36, 56, 57]. Our results showed cloxacillin’s MIC and MBC values against all strains ranged from 0.5 to 512 μg/mL and 1 to 1024 μg/mL, respectively. MIC_90_ of cloxacillin was 256 μg/mL, similar to Omara et al. [58]. Our results showed that cefazolin’s MIC and MBC values against all strains ranged from 0.125 to 128 μg/mL and 1 to 512 μg/mL, respectively. Finally, our results showed that clindamycin’s MIC and MBC values against all strains ranged from 0.125 to 64 μg/mL and 4 to >1024 μg/mL, respectively. This study found a vancomycin-resistant *S*. *epidermidis* (VRSE) strain with MIC equal to 32 μg/mL.

Antibiotics were the main solution that allowed humans to survive bacterial infections that were lethal in the past, but the emergence of bacterial drug resistance caused challenges to public health [59–61]. On top of this, it has been found that sub-MIC values of antimicrobial agents can change bacteria’s physiological and biochemical functions, such as biofilm induction during antimicrobial agent’s chemotherapy [62–64]. Because bacteria are exposed to sub-MICs of antibiotics at the beginning and end of a dosing regimen, between doses, or continually during low-dose treatment, the process of antibiotic-induced biofilm formation may have therapeutic importance [65]. Accordingly, this study evaluated the effect of sub-MIC values of cloxacillin, cefazolin, clindamycin, and vancomycin on MRSE strains using the microtiter plate biofilm quantification technique as well as the expression of biofilm-associated genes by RT-PCR.

The most notable result of the present study was the ability of different subinhibitory concentrations of cloxacillin, cefazolin, and clindamycin to induce biofilm production in the tested *S*. *epidermidis* strains. In total, the OD values of strains were in the range of 0.09–0.95, 0.05–0.86, and 0.06–1 toward cloxacillin, cefazolin, and clindamycin, respectively. In this regard, sub-MIC values of cloxacillin found the maximum enhancement in biofilm production in the presence of 64 to 0.0312 μg/mL of cloxacillin (Fig 2). Regarding ATCC 35984, all sub-MIC concentrations of cloxacillin significantly impacted biofilm formation and maximum OD increase was found at concentration of 4 μg. Consistent with our results, Mirani et al. [66] found that oxacillin facilitates *S*. *aureus’s* adhesion and biofilm production exclusively on glass surfaces. In another study, Qu et al. [67] found that biofilm of some CoNS is increased by oxacillin in a concentration-dependent manner. Besides, Weiser et al. [68] found that at sub-inhibitory concentrations, oxacillin exhibited significant biofilm-inducing activity in *S*. *epidermidis*. In a study, Cerca et al. [69] found that exposure to sub-MIC of dicloxacillin can induce polysaccharide production in *Staphylococcus haemolytics*. In their research, *S*. *epidermidis* biofilms formed in the presence of a sub-MIC concentration of dicloxacillin produced significantly smaller amounts than the control. Besides, in a study by Mirani et al. [70], they found that biofilm formation was induced in most of the methicillin-resistant *S*. *aureus* (MRSA) strains after 24 h of exposure to the sub-MIC concentration of oxacillin. In a study, it has been found that exposure to sub-MIC values of methicillin led to a dramatic enhancement in biofilm production in *S*. *aureus* USA300 and USA500 that was mediated by autolysis activity of *atl*, showing a genetic mechanism that causes bacterial lysis to liberate eDNA that can enhance biofilm production [71].

Our findings are the first to report that sub-MICs of clindamycin induce bacterial biofilm formation in *S*. *epidermidis*. In this regard, maximum biofilm induction was found in ATCC 35984 by approximately 9-fold after exposure to 1 μg/mL clindamycin. Clindamycin, a protein synthesis inhibitor used to treat gram-positive bacterial infections, causes *S*. *aureus* to produce virulence factors [29], and biofilm formation, but these effects have not been reported in *S*. *epidermidis*.

*S*. *epidermidis* infections associated with devices usually require vancomycin for treatment [68]. Several previous studies investigated the effects of sub-MICs of vancomycin on *S*. *epidermidis* biofilm formation, and they found little or no effect of sub-MIC vancomycin on *S*. *epidermidis* biofilm formation that accordance with our results [68, 72–74]. For example, in Cerca et al. [72] study, sub-MIC of vancomycin had a minor effect on bacterial adherence and biofilm formation. Current reports in some kinds of biofilm-producing *S*. *epidermidis* strains that encounter vancomycin has found that the biofilm matrix maybe is not be fully produced under standard laboratory conditions, and they are only expressing the biofilm matrix in the presence of additional environmental stimuli like the serum. Hence, this notion may be also considered for S. epidermidis strains against sub-MIC vancomycin values in our study. However, in a study by Cargill et al. [75], a range of responses was observed including increased biofilm density at vancomycin concentrations approaching the MIC against *S*. *epidermidis* isolates from prosthetic orthopedic infections which is opposite to our findings. What is important about the biofilm production ability in the face of antibiotics is that this capability can be directly related to the level of resistance to the desired antibiotic. Evolutionarily, bacteria change phase when exposed to an unfavorable factor and signal, or in other words, acquire the ability to adapt to those unfavorable conditions [76, 77]. According to the reports, biofilm production can be one of the adaptations [78]. The point that can be made at first glance about the difference between our results and others regarding the production of biofilm with vancomycin is the latter case, for example in some other areas, VRSE has been previously reported [79, 80], while in Iran VRSE only recently reported andit needs much time to appear VRES with induced biofilm effect toward MIC and/or sub-MIC values of vancomycin. As a result, it may still take time for *S*. *epidermidis* isolates in Iran to be stimulated to form a biofilm upon exposure to vancomycin.

Inducing biofilm formation by oxacillin can describe some treatment failures, as flucloxacillin is usually administrated to treat staphylococcal infections. However, the mechanism of subinhibitory concentrations oxacillin inducing biofilm production of staphylococci still needs further study. It has been found that bacteria exposed to antimicrobial agents show some stress reactions, resulting in biofilm production, dependent on the dosage and nature of the antimicrobial agents and bacterial strain [81]. It has been found that staphylococcal strains may not form biofilm in some conditions but can turn biofilm producers into new conditions because of changes in environmental signals [82]. In usual conditions, biofilm formation in *S*. *epidermidis* could be induced by long-term exposure to external factors like high NaCl and glucose levels via stimulation *ica* locus [82]. The ability of the sub-MIC concentrations of anti-bacterial cell wall agents such as beta-lactams and also anti-protein synthesizing agents to act as signal molecules was demonstrated by the specificity of the biofilm induction phenotype for cefazolin, cloxacillin, and clindamycin, corroborating this conclusion. In the current study, the effect of sub-MIC concentrations of cloxacillin, cefazolin, clindamycin, and vancomycin on the expression of the *atlE* and *icaA* were evaluated for the selected strains, and the results showed that the expression of *atlE* and *icaA* were upregulated in examined *S*. *epidermidis* strains against cloxacillin, cefazolin, and clindamycin at some concentrations. In particular, *atlE* and *icaA* were upregulated 0.062 to 1.16 and 0.078 to 1.48 folds, respectively, for cloxacillin, 0.11 to 0.8, and 0.1 to 1.3 folds for cefazolin, 0.18 to 0.98, and 0.19 to 1.4 folds, respectively, for clindamycin. These data imply that antibiotic-induced biofilm development may depend on increased poly-N-acetylglucosamine (PNAG) synthesis, a significant component of the *S*. *epidermidis* biofilm matrix, that mediates several biofilm-related activities including intercellular adhesion and resistance to killing by antimicrobial peptides and phagocytes [83, 84]. Strains obtained from infections have a genetic region that encodes the generation of PNAG so-called ica, which suggests that PNAG plays a function in human infections [85, 86]. Consistent with our results, Mirani et al. [66] found showed the expression of the *icaA* in *S*. *aureus* and subsequent biofilm formation activated by oxacillin. Schilcher et al. [29] found that sub-MIC values of clindamycin stimulated a stress reaction in *S*. *aureus* through the alternative sigma factor B that up-regulated the expression of the biofilm-associated genes such as *fibronectin-binding proteins A (FnbA)*, *fnbB*, *atlA*, *accessory gene regulator A (agrA)*, *lrgA*, and *phenol-soluble modulin (psm)*. Their findings also showed that sub-MIC values of clindamycin change the capacity of *S*. *aureus* to generate biofilm and change the structure of the matrix biofilm to a higher eDNA rate. In some studies, subinhibitory concentrations of oxacillin, and clindamycin did not affect *ica* expression in *S*. *epidermidis* even though these antibiotics induced biofilm [23].

One reason could be that in staphylococci, biofilm production is in some cases non-*ica*-dependent, and, these antibiotics may have induced biofilm from the *ica*-independent pathway. Finally, our results indicate that the amount of biofilm formation induced by sub-MIC antibiotics is inversely proportional to the amount of biofilm produced by the *S*. *epidermidis* test strain in the absence of antibiotics. These results are consistent with those of Kaplan et al. [86] and Pérez-Giraldo et al. [87], who showed that was inversely proportional to the amount of biofilm formation in the absence of antibiotics. Besides, our results show that antibiotic-induced biofilm formation in *S*. *epidermidis* is antibiotic- and strain-dependent. In this regard, sub-MIC cloxacillin, cefazolin, and clindamycin induced biofilm formation in some strains, but vancomycin did not. Also, antibiotic induced biofilm formation in ATCC 35984, MRSE4, MRSE 5, MRSE 10, and MRSE 13 was increasingly found.

Collectively, taking our findings together show that the observed biofilm induction by subinhibitory concentrations of cloxacillin, cefazolin, and clindamycin may be due to an *ica*-dependent pathway, however, more experiments, as well as the action mechanism studied with antibiotic-sensitive and -resistant *S*. *epidermidis* isolates, are needed to further confirm this.

## 5. Conclusion

Due to the inappropriate use, low concentrations of antimicrobial agents have commonly existed at the site of infection. The emerging body of reports shows that exposure to sub-MIC values of antibiotics causes influences the phenotype of bacterial cells, such as inducing biofilm formation. In summary, the results of the present study demonstrate that sub-MIC concentrations of antibiotics significantly induce biofilm formation in MRSE strains, and the current study is the first that found the effect of sub-MIC of cloxacillin, cefazolin, and clindamycin on the induction of biofilm formation in *S*. *epidermidis*. Our results show that the amount of antibiotic-induced biofilm formation is antibiotic-specific and is inversely proportional to the amount of biofilm produced by the test strain. The findings reported here could be used by physicians to appropriate antibiotic administration. Therefore, the condition and molecular mechanisms contributing to low values of antibiotics to inducing biofilm formation merit further study. Correct administration of antimicrobial agents is necessary to decrease the risk of the emergence of resistant bacteria and induce biofilm formation. Accordingly, current findings will help establish the medical application to guide antibiotic therapy that would prevent biofilm induction. Future studies are needed to investigate the induction of biofilm formation by exposure to sub-MIC of other antibiotics in MRSE.

## Supporting information

S1 File (RAR)
